# Broadly Protective Strategies Against Influenza Viruses: Universal Vaccines and Therapeutics

**DOI:** 10.3389/fmicb.2020.00135

**Published:** 2020-02-07

**Authors:** Olivia A. Vogel, Balaji Manicassamy

**Affiliations:** ^1^Department of Microbiology, The University of Chicago, Chicago, IL, United States; ^2^Department of Microbiology and Immunology, The University of Iowa, Iowa City, IA, United States

**Keywords:** influenza, universal influenza vaccine, vaccine, influenza therapeutics, immunization

## Abstract

Influenza virus is a respiratory pathogen that can cause disease in humans, with symptoms ranging from mild to life-threatening. The vast majority of influenza virus infections in humans are observed during seasonal epidemics and occasional pandemics. Given the substantial public health burden associated with influenza virus infection, yearly vaccination is recommended for protection against seasonal influenza viruses. Despite vigilant surveillance for new variants and careful selection of seasonal vaccine strains, the efficacy of seasonal vaccines can vary widely from year to year. This often results in lowered protection within the population, regardless of vaccination status. In order to broaden the protection afforded by seasonal influenza vaccines, the National Institute of Allergy and Infectious Diseases (NIAID) has deemed the development of a universal influenza virus vaccine to be a priority in influenza virus vaccine research. This universal vaccine would provide protection against all influenza virus strains, eliminating the need for the yearly reformulations of seasonal influenza vaccines. In addition to universal influenza vaccine efforts, substantial progress has been made in developing novel influenza virus therapeutics that utilize broadly neutralizing antibodies to provide protection against influenza virus infection and to mitigate disease outcomes during infection. In this review, we discuss various approaches toward the goal of improving influenza virus vaccine efficacy through a universal influenza virus vaccine. We also address the novel methods of discovery and utilization of broadly neutralizing antibodies to improve influenza disease outcomes.

## Introduction

Influenza viruses are a significant public health burden worldwide, causing yearly epidemics and occasional pandemics. Infection with influenza virus causes acute upper respiratory disease in humans that can potentially lead to hospitalization or death. In addition to the morbidity and mortality associated with influenza virus infection, the yearly economic burden of influenza virus infections in the United States is estimated to be around $11.2 billion (Putri et al., 2018). Given the considerable impact of influenza virus infection in communities worldwide, significant attention has been focused on preventing influenza virus infection and spread of the virus through vaccination within the population as well as with other public health measures.

Influenza viruses are members of the *Orthomyxoviridae* family of viruses, which are characterized by segmented, negative sense, single-stranded RNA genome. Of the influenza virus types, influenza A and B are the only types that are known to cause disease in humans. In addition to humans, influenza A viruses can infect a broad variety of species including pigs, horses, and birds (Webster et al., 1995). In nature, influenza A viruses are maintained in water fowls, which are the main reservoir for influenza A (Webster et al., 1995). Influenza A viruses can be further classified into different subtypes based on the two major viral surface glycoproteins, hemagglutinin (HA) and neuraminidase (NA) (Fields et al., 2013). For influenza A viruses, there are 18 known HA subtypes that fall into two phylogenetic groups (Group 1 or Group 2); like HA, the 11 NA subtypes also fall within two phylogenetic groups. These phylogenetic groups are composed of viruses that are derived from a common ancestor. Unlike influenza A viruses, the diversity of influenza B viruses is limited and is categorized into two lineages, B/Yamagata and B/Victoria (Rota et al., 1990). Despite the limited diversity, influenza B viruses evolve to escape immunity and remain in circulation in humans; thus, necessitating yearly updates of the influenza B virus strains included in the seasonal vaccine.

## Immunological Responses to Influenza Virus Infection

Influenza viruses predominantly infect and replicate in the epithelial cells lining the upper respiratory tract. Viral infection is initiated by the binding of viral surface glycoprotein HA to host sialic acid residues followed by internalization of the virus through endocytosis (Fields et al., 2013). Subsequently, the fusion of the viral membrane with the endosomal membrane releases the viral genomic RNA into the cytoplasm, and the RNA is then imported into the nucleus for replication (Fields et al., 2013). The initial innate immune responses against influenza virus infection are activated by the sensing of viral RNA by pattern recognition receptors such as the retinoic acid-inducible gene-I (RIG-I) and Toll-Like Receptor 7 (TLR7) (Iwasaki and Pillai, 2014). Additional innate sensing pathways also contribute to robust innate responses against influenza virus infection (Iwasaki and Pillai, 2014). Ultimately, the activation of these innate sensing pathways leads to the production of interferon and cytokines/chemokines critical for efficient activation of adaptive immune responses (B- and T-cell responses) that help control and clear infection.

Studies in humans and mice demonstrate the importance of T-cell responses in clearing primary influenza virus infection and mounting robust recall responses in subsequent infection. The importance of T-cell responses was highlighted by a study following 342 healthy adults in the UK during the 2009 H1N1 pandemic, which determined that illness was less severe in individuals with higher frequencies of pre-existing T cells to conserved CD8 epitopes (Sridhar et al., 2013). The importance of CD8^+^ T cells during influenza virus infection was further highlighted in adoptive transfer experiments in which mice were given CD8^+^ effector cells. After infection, viral replication was reduced in the lungs of recipient mice compared to mice that did not receive CD8^+^ T cells (Yap et al., 1978; Lukacher et al., 1984; Hamada et al., 2009, 2013). Additionally, mice receiving CD8^+^ T cells also displayed increased recruitment of NK cells, macrophages, and B cells after infection (Hamada et al., 2013). These results further highlight the importance of mounting CD8^+^ immune responses during infection. More recently, CD4^+^ T cells have also been shown to have an important role in clearing influenza infection, with the lack of CD4^+^ T cells correlating with reduced viral clearance (Belz et al., 2002). As with CD8^+^ T cells, adoptive transfer of CD4^+^ memory T cells in mice was associated with greater protection during influenza infection (McKinstry et al., 2012).

The mucosal antibody response is an important feature in determining the ability of the host to efficiently clear viral infections. Mucosal antibody responses are more effective in preventing subsequent infection rather than primary viral infection. The three main immunoglobulin (Ig) isotypes induced during influenza infection are IgG, IgA, and IgM. Secretory IgA antibodies are generated early during infection and can act as an indicator of acute influenza infection (Rothbarth et al., 1999). Additionally, secretory IgA antibodies have been associated with greater protection in the upper respiratory tract while also providing cross-reactive protection against different influenza virus strains (Tamura et al., 1990; Asahi et al., 2002; Renegar et al., 2004; Ainai et al., 2013). IgM antibodies are also generated during primary infection and have been shown to have a role in complement mediated virus neutralization (Rothbarth et al., 1999; Fernandez Gonzalez et al., 2008). In contrast to serum IgA antibodies, serum IgG antibodies are associated with protection in the lower respiratory tract as well as providing strain-specific protection (Tamura et al., 1990).

## Current Seasonal Influenza Vaccines

The composition of seasonal influenza virus vaccines is based on the strains currently propagating in the human population. Presently, there are two influenza A virus strains (H1N1 and H3N2) and two influenza B virus lineages circulating in humans, with only one influenza B strain from each type circulating as the predominant strain during influenza season. However, as it is impossible to predict the predominant strain for a given season, the majority of the current seasonal influenza vaccines are composed of all four strains. The selection of influenza strains for incorporation into seasonal vaccines is based on surveillance of circulating strains by WHO influenza centers as well as on an assessment of which strains will likely become the predominant strain in human populations (Gerdil, 2003). Once the vaccine strains have been selected by the WHO committee, seasonal vaccine production can begin and requires roughly 6 months for the commonly used inactivated vaccine to be produced and distributed (Gerdil, 2003).

There are four types of seasonal influenza virus vaccines currently licensed for use in humans: inactivated, live attenuated, recombinant protein, and cell-based vaccines. A vast majority of the influenza virus vaccines administered in humans are split inactivated vaccines, which are produced in embryonated chicken eggs. For split inactivated vaccines, vaccine strains are individually grown in the allantoic cavity of embryonated chicken eggs and inactivated by treatment with formalin or β-propriolactone (Gerdil, 2003). Once purified, virus particles are then split using ether and detergent to reduce the level of viral ribonucleoproteins, which cause reactogenicity at the site of injection (Gerdil, 2003). The individual vaccine components are mixed in a standardized manner to ensure an inclusion of 15 μg of HA per strain (Sridhar et al., 2015). Inactivated vaccine is administered intramuscularly to stimulate the systemic immune response, producing mainly IgG antibodies and low amounts of IgA antibodies (Clements et al., 1986). Similar to inactivated vaccines, live attenuated influenza vaccines (LAIVs) are grown in embryonated eggs. LAIV strains are generated using a reverse genetics approach to incorporate HA and NA genes from circulating influenza strains into a cold-adapted, attenuated influenza virus backbone (Sridhar et al., 2015). Cold adaption of the LAIV backbone ensures that the replication of the vaccine strain does not occur at temperatures above 33°C, allowing for replication in the upper respiratory tract but not in the lower respiratory tract (Sridhar et al., 2015). LAIV, which is administered intranasally to mimic natural infection, stimulates robust IgA and IgG responses in the upper respiratory tract (Clements et al., 1986). LAIV has also been shown to elicit T-cell mediated responses in vaccinated children (He et al., 2006; Hoft et al., 2011). While both inactivated vaccine virus strains and LAIV viruses are grown in embryonated eggs, this poses a challenge in the event of egg shortages and for immunization of individuals with egg allergies. Recombinant protein vaccines and cell culture-based influenza vaccines have been developed to overcome limitations of egg-based vaccines. Currently, there is only one recombinant vaccine, named Flublok^®^, approved for use by the US Food and Drug Administration (FDA). Flublok^®^ still contains HA protein antigens representing the selected influenza strains for the current season, but it is produced in insect cells (Cox and Hollister, 2009). In contrast to embryonated egg-based vaccines, manufacturing of Flublok^®^ takes approximately 2 months and can be administered in individuals with egg allergies, providing an advantage over traditional seasonal egg-based vaccines (Cox and Hollister, 2009). In regard to cell-based vaccines, Flucelvax^®^ is a licensed quadrivalent inactivated vaccine that is grown in a mammalian cell line and can avoid any potential egg-based mutations (Lamb, 2019). As with Flublok^®^, Flucelvax^®^ can be administered to individuals with egg allergies and requires a shorter production time than egg-grown vaccines.

## Challenges for Current Seasonal Vaccines

Yearly vaccination with split, inactivated influenza vaccines is still one of the most popular and efficient means of protection against seasonal influenza viruses. Prior to 2012, influenza vaccines were trivalent and contained only one influenza B virus in the vaccine. This often resulted in inadequate protection against influenza B virus, due to mismatches between the circulating influenza B virus lineage and the influenza B virus lineage chosen for vaccine incorporation (Tisa et al., 2016). To avoid issues with influenza B virus lineage mismatches, a quadrivalent inactivated vaccine that contains both influenza B virus lineages was developed (Tisa et al., 2016). Despite these improvements to the seasonal influenza virus vaccines, the effectiveness of seasonal influenza vaccines can vary greatly, depending on how closely the vaccine strains match the circulating strains. In fact, there are several instances in the recent past where seasonal vaccines failed to provide protection against antigenically drifted strains (David et al., 2005; Halloran et al., 2007; Skowronski et al., 2007). Furthermore, yearly vaccines do not provide protection against novel influenza virus strains introduced from zoonotic reservoirs, causing significant morbidity and mortality due to lack of immunity in the general population.

The types of immune responses elicited by different seasonal influenza vaccines also impact the degree and the longevity of protection. As mentioned previously, inactivated vaccines administered intramuscularly elicit production of serum IgG antibodies but fail to induce antibody and T-cell responses in the respiratory mucosa. As such, this type of vaccine provides strain-specific protection but can leave individuals vulnerable to infection by new variant strains. Preclinical studies indicate that intranasal administration of inactivated vaccines induces robust responses in the respiratory mucosa. LAIV administered intranasally closely mimics natural infection and stimulates robust IgA and IgG antibody responses; however, this vaccine failed to provide adequate protection against influenza A(H1N1)pdm09 during the 2013–2014 and 2015–2016 seasons (Grohskopf et al., 2016, 2018). This inadequate protection prompted the Advisory Committee on Immunization Practices (ACIP) to recommend against administration of this vaccine for the 2016–2017 and 2017–2018 seasons (Grohskopf et al., 2016, 2017). Later, the manufacturer determined that the reduced protection associated with the LAIV was most likely due to reduced replicative fitness of the A/California and A/Bolivia (H1N1)pdm09 LAIV strains (Ambrose et al., 2016). Currently, the quadrivalent LAIV has incorporated a new influenza A(H1N1)pdm09-like virus that provides better protection and is recommended again for use (Grohskopf et al., 2018). While the LAIV has been demonstrated to provide robust protection in younger children, responses to LAIV vaccination in preimmune adults are likely limited due to immune-mediated restriction of LAIV replication prior to induction of effective immunity.

A vast majority of seasonal influenza vaccines are manufactured in eggs. Occasionally, during vaccine production, egg adaptive mutations can arise that alter the antigenicity of the virus and, as a result, can reduce vaccine effectiveness against circulating strains. H3N2 strains in particular have been associated with increased egg-based mutations that result in reduced antigenicity of the vaccine strain, negatively impacting the protection afforded by the vaccine (Skowronski et al., 2014; Wu et al., 2017, 2019; Zost et al., 2017). Consequently, egg grown vaccines must be monitored to ensure that the vaccine strain incorporated into the vaccine matches the seed strain chosen at the start of manufacturing. The risk of adaptive mutations and reduced antigenicity is mitigated in recombinant protein vaccines, which are grown in insect cells, or by producing the vaccine strains in mammalian cell lines. However, the cost associated with large-scale production of cell-culture grown vaccine strains remains high as compared to ones produced in eggs (Barr et al., 2018).

Another alternative to egg grown vaccine approaches involves the use of DNA and mRNA vaccines. While none are currently approved for use, DNA and mRNA vaccines can be manufactured synthetically, allowing for quicker production times than egg and cell-culture grown vaccines (Stachyra et al., 2014; Kanekiyo et al., 2019) Furthermore, DNA and mRNA vaccine approaches do not induce the anti-vector immune responses that can be elicited with viral vector-based vaccine approaches (Kanekiyo et al., 2019). DNA vaccines have also shown promise as a method of vaccine priming (Ledgerwood et al., 2011). In fact, several of the universal vaccination methods described in this review utilize DNA prime-boost vaccination to improve immune responses. Likewise, mRNA-based vaccines show potential as an alternative vaccination approach. An example of an mRNA-based approach includes RNActive^®^ vaccines. RNActive^®^ vaccines are mRNA vaccines complexed with protamine, which allow for the mRNA to acts as a “self-adjuvant” through interaction with TLR7 (Kallen et al., 2013). An RNActive^®^ vaccine encoding the full-length PR8 HA has been shown to induce IgG antibodies against PR8 HA and provide protection against homologous PR8 viral challenge (Petsch et al., 2012). Furthermore, transfer of serum from PR8 HA mRNA-vaccinated mice to unvaccinated recipient mice provided protection against PR8 infection (Petsch et al., 2012). Together, these different vaccination approaches have the potential for improving upon yearly influenza virus vaccines as well as in the development of universal influenza virus vaccines.

An additional consideration for seasonal influenza vaccines is the ability to provide protection among older adults, who are often at higher risk for influenza infection. The usual dosage of 15 μg HA antigen per vaccine strain in seasonal inactivated vaccines is insufficient to induce protection in older adults. To address this concern, a new vaccine named Fluzone^®^ was developed to improve serum antibody responses in older adults. Fluzone^®^ is a trivalent, inactivated split-virus vaccine that contains 60 μg of HA for each influenza strain included in the vaccine (Robertson et al., 2016). This dosage is four times that of standard inactivated vaccines and has been shown to increase protection in older adults, providing a promising alternative for this high-risk group (Robertson et al., 2016).

The most significant challenge for the seasonal influenza vaccine production strategy involves ensuring that the vaccine strains match the dominant circulating strains in the population. There have been significant efforts to improve upon methods of monitoring and identifying newly evolved influenza strains worldwide, including the incorporation of modeling techniques such as antigenic cartography (Ampofo et al., 2015). Despite these efforts, the screening process is imperfect, and as result, it is not unusual to see the emergence of a variant strain after selection of another strain for vaccine production. Neutralizing antibodies induced upon vaccination predominantly target the highly immunogenic head domain of HA and provide potent protection against matching strains by blocking virus attachment to cells (Knossow et al., 2002). Consequently, these highly immunogenic regions in the head domain are prone to high mutation rates due to immune selection pressure from neutralizing antibodies. In a circulating strain, accumulation of mutations in the head domain over time leads to changes in antigenic properties (antigenic drift) and can render vaccine-induced immunity ineffective against them. In addition, these variant strains can cause epidemics in populations lacking immunity to the newly evolved strain (Fields et al., 2013). Antigenic drift is the major driving force behind the need to reformulate influenza vaccines each year and poses a significant challenge for vaccine development. Furthermore, seasonal influenza virus vaccines fail to provide protection against the novel strains transmitted from zoonotic reservoirs.

**Figure 1:**
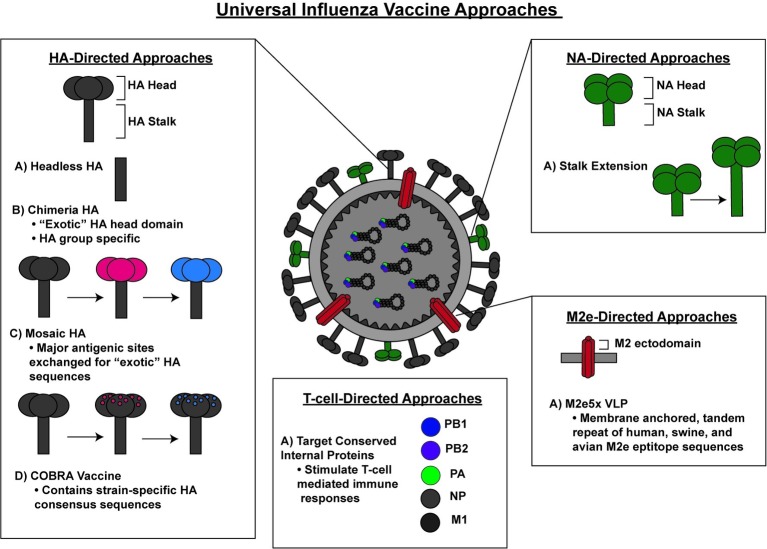
A schematic representation of various preclinical approaches for a universal influenza vaccine.

Given the considerable public health consequences associated with a lack of sufficient protection against seasonal and pandemic influenza stains, the National Institute of Allergy and Infectious Diseases (NIAID) has prioritized the development of a universal influenza vaccine that can afford protection against a broad variety of influenza viruses (Erbelding et al., 2018). Currently, the majority of universal influenza vaccine research efforts are aimed at inducing immunity against the highly conserved regions on the influenza surface proteins or internal proteins of influenza viruses as means to induce universal protection against all influenza virus strains (Figure 1).

## Universal Influenza Vaccine Approaches

### Hemagglutinin-Directed

The influenza virus surface protein HA is one of the major targets of the immune system. HA is synthesized as a precursor HA0 that is proteolytically processed into HA1 and HA2 subunits, which remain attached through a disulfide bond. Three HA molecules form a trimeric rod-shaped molecule that is composed of a long fibrous stem formed by the HA2 trimer and a globular head formed by the HA1 trimer (Fields et al., 2013). The globular head domain of HA contains the receptor binding pocket, which facilitates virus attachment to the host cell surface sialic acids (Wilson et al., 1981; Rogers et al., 1983). Once the bound virus is internalized through endocytosis, HA undergoes conformational changes under low pH conditions that allow HA2 to initiate the fusion of the viral and endosomal membranes (Skehel et al., 1982; Bullough et al., 1994). This process enables the release of the viral genomic contents into the cytoplasm. The HA1 region is highly immunogenic, more flexible to accommodate mutations than HA2 and displays a high degree of variability among influenza virus strains. Conversely, the HA2 region is highly conserved among different influenza virus subtypes and is more structurally constrained in its ability to accommodate mutations.

As described above, the antigenic variation of the HA1 domain and its resulting antigenic drift is the major reason for reformulating seasonal influenza vaccines with new strains every year. To address this challenge, several vaccine strategies based on conserved sequences in HA have been developed. Once such approach involves a novel computational-based antigen design methodology. This approach, termed computationally optimized broadly reactive antigen (COBRA), was used to generate a consensus H5 HA sequence by aligning 129 unique HA sequences from clade 2 H5N1 viruses isolated from 2004 to 2006 (Giles and Ross, 2011). Following alignment, the most common amino acid at each position was selected to generate a consensus sequence for the COBRA H5 HA (Giles and Ross, 2011). This newly generated COBRA H5 HA was confirmed to exhibit normal HA activity, including receptor binding and particle fusion (Giles and Ross, 2011). The efficacy of this COBRA H5 HA was evaluated in virus-like particle (VLP) based vaccination and challenge studies (Giles and Ross, 2011). Following vaccination of mice and ferrets, the COBRA H5N1 VLP vaccines induced increased hemagglutinin inhibition titers and provided enhanced protection against lethal challenge with different clade 2 H5N1 viruses when compared to a VLP vaccine containing an HA derived from a primary isolate (Giles and Ross, 2011). The improved efficacy of COBRA vaccine approaches have been supported by studies in a non-human primate model (cynomolgus macaques) with the COBRA HA H5N1 VLPs (Giles et al., 2012). The sera from vaccinated macaques demonstrated hemagglutinin inhibition titers against a wider range of H5N1 strains than animals vaccinated with contemporary H5N1 HA VLPs (Giles et al., 2012). In addition, vaccinated macaques were protected against viral challenge as well as exhibiting reduced lung inflammation (Giles et al., 2012). Following the same methodology used for the generation of COBRA HA H5N1 VLPs, COBRA HA H3N2 and COBRA HA H1N1 VLPS have been generated using consensus sequences from both modern and historical H3N2 and H1N1 strains. These COBRA VLP vaccines provided broad protection against a wide range of H3N2 and H1N1 strains (Carter et al., 2016; Wong et al., 2017). Recently, the COBRA H1 and H3 antigens, along with the AF03 adjuvant (squalene-in-water emulsion), induced protective responses in ferrets, with ferrets exhibiting decreased viral shedding (Allen et al., 2018). By generating more broadly reactive HAs, the COBRA approach provides an intriguing solution to overcome the challenges posed by antigenic drift of circulating strains, as COBRA-based seasonal vaccine has the potential to provide protection even during mismatch. However, as protection afforded by COBRA vaccines is only against specific HA subtypes, COBRA vaccines still fall short of serving as a true universal influenza vaccine.

To generate broadly protective immune responses against multiple influenza virus strains, several research groups developing universal influenza vaccines have targeted the highly conserved stalk domain of HA. Antibodies directed against the HA stalk domain have been shown to be protective in both mice and humans (Okuno et al., 1993; Throsby et al., 2008; Ekiert et al., 2009). The stalk-specific antibodies provide protection by various mechanisms, including by directly preventing HA-mediated fusion or by inducing antibody-dependent cellular cytotoxicity (ADCC; Ekiert et al., 2009; DiLillo et al., 2014; He et al., 2016). Importantly, antibodies directed toward the HA stalk domain have been shown to provide cross-reactive protection against multiple influenza virus strains (Throsby et al., 2008; Sui et al., 2009). However, this cross-reactive protection is typically restricted to HAs from the same group, Group 1 HA-specific stem-directed antibodies are unable to neutralize infection by viruses carrying Group 2 HAs (Sui et al., 2009).

A major hurdle in developing effective vaccine strategies targeting the HA stalk domain is overcoming the poor immunogenicity of the HA stalk domain, as in the context of full-length HA, antibodies are mainly produced toward the highly immunogenic HA head domain (Vareckova et al., 1993). Studies performed in the 1980s with monoclonal antibodies that were cross reactive against H1 and H2 subtypes of HA demonstrated that a vaccine approach targeting the stem region of HA can provide broad protection and overcome the limitation of antigenic drift (Graves et al., 1983). This was further confirmed in a 1996 study in which mice were immunized with cells overexpressing an HA that lacked the globular head domain (Sagawa et al., 1996). Following challenge with H1N1, mice that were immunized with the headless HA exhibited increased survival compared to mice immunized with full length HA expressing cells (Sagawa et al., 1996). These studies underscore the potential immunogenicity of the HA stalk in the absence of the HA head domain.

In recent years, vaccine approaches have attempted to enhance exposure of the HA stalk domain to the immune system using HAs that lack the dominant head domain (Steel et al., 2010; Impagliazzo et al., 2015). These “headless” HAs were incorporated onto VLPs for vaccination (Steel et al., 2010; Impagliazzo et al., 2015). Nanoparticles have also been used as a method to improve HA stalk exposure during vaccination, using either HAs lacking the head domain or full-length HAs (Kanekiyo et al., 2013; Yassine et al., 2015). Vaccination in mice and ferrets demonstrated that these nanoparticle-based approaches provided cross-reactive protection following viral challenge with different influenza A virus strains (Kanekiyo et al., 2013; Yassine et al., 2015). Interestingly, when ferrets were immunized with the nanoparticle vaccine containing a full-length HA fused to ferritin, both HA stem and receptor binding site-specific antibodies were detected, demonstrating the utility of this approach in generating more broadly immunogenic HA-based vaccines (Kanekiyo et al., 2013).

Another method of improving exposure of the HA stalk domain involves the generation of chimeric HAs (cHA) that express the head domain from one virus strain and the stalk domain of another. This method involves sequential immunization with constructs that express the same stalk domain but different “exotic” head domains, thereby specifically stimulating stalk-directed antibody responses to induce broader protection than strain-specific HA head directed antibodies generated by seasonal vaccines (Hai et al., 2012; Pica et al., 2012; Krammer et al., 2013; Margine et al., 2013; Nachbagauer et al., 2017; Liu et al., 2019). Sequential vaccination of mice with cHA containing the same H1 stem but different heads demonstrated protection against challenge from Group 1 viruses but not Group 2 viruses (Krammer et al., 2013). In a recent preclinical study, ferrets were immunized with a LAIV virus expressing a cHA composed of an H8 head and a H1 stalk (cH8/1) as well as the N1 NA from the 2009 H1N1 pandemic virus (Nachbagauer et al., 2018). Subsequently, ferrets received a boost with a split virus vaccine containing a cHA with a H5 head and H1 stalk (cH5/1 IIV) (Nachbagauer et al., 2018). Ferrets vaccinated with cHA demonstrated greater protection against the pandemic H1N1 challenge as compared to ferrets immunized with two doses of a seasonal trivalent influenza vaccine, demonstrating improved protection elicited by the cHA vaccination approach (Nachbagauer et al., 2018). Despite the lack of protection across different HA groups, the cHA approach shows promise for human vaccination and has been recently evaluated in human clinical trials. The outcome of the human clinical trials is still being evaluated (Bernstein et al., 2019).

Recently, the cHA vaccine has been further improved upon through the development of mosaic HAs (mHA), in which only the major antigenic sites in the HA head domain are exchanged with “exotic HA” sequences (Broecker et al., 2019a). This strategy was developed to generate antibodies against both the stalk domain and epitopes in the head domain that fall outside the major antigenic sites (Broecker et al., 2019a). Sequential vaccination with inactivated mHA viruses induced cross-reactive antibodies to both the stalk domain and the head domain (Broecker et al., 2019a). Additionally, transfer of sera from vaccinated mice into naïve mice provided protection against viral challenge with reassortment viruses containing PR8 internal proteins with HA and NA from different H3N2 strains as compared to the seasonal inactivated quadrivalent vaccine (Broecker et al., 2019a). These results indicate that the mHA approach also has potential to provide broader protection than current seasonal vaccines.

An important challenge for HA stalk-directed vaccines is whether these vaccine strategies are capable of providing protection against strains from both HA groups. As described above, HA stalk-directed antibodies often provide HA group-specific protection. While the ability to provide protection against a broad range of influenza virus strains within a given HA subtype is a vast improvement over approved seasonal vaccines, this still falls short of providing universal influenza virus protection. Another important consideration involves providing protection for both influenza A and B virus strains. While the HA stalk-directed approaches described above demonstrate protection against influenza A viruses, protection against influenza B strains is not always addressed. A truly universal vaccine should be able to provide protection against both influenza A and B strains. Therefore, current universal vaccine research should be cognizant of the need to elicit protection for both types of influenza viruses. Antibodies targeting the HA stalk domain have been shown to be protective against influenza B strains, with the human monoclonal antibody CR9114 demonstrating protection against lethal challenge from both influenza A and B strains (Dreyfus et al., 2012). Recently, the mHA approach was used in an attempt to generate a universal influenza B vaccine that could provide protection against a wide range of influenza B virus lineages (Sun et al., 2019). To generate the influenza B mHA, the major antigenic sites were replaced with “exotic” influenza A sequences (Sun et al., 2019). Immunization of mice involved a DNA primer followed by two mHA protein boosts (Sun et al., 2019). Importantly, mice vaccinated with the influenza B mHA demonstrated improved survival following lethal challenge with different influenza B strains (Sun et al., 2019). While this study does not provide evidence for protection against influenza A strains, it further serves to highlight the potential for vaccination approaches that improve upon the breadth of protection provided by the current seasonal vaccination strategies. It would be interesting to examine whether combining influenza A mHAs and influenza B mHAs into a single vaccine might provide even greater protection for both influenza A and B strains than the current inactivated quadrivalent vaccine.

### Neuraminidase-Directed

The second major surface protein for influenza A virus is the NA protein, which has an important role in facilitating virus release from the host cell. The sialidase or neuraminidase activity of NA helps cleave the terminal sialic acids from glycans and thereby facilitate virion release from infected cells (Fields et al., 2013). NA has been an important target for the development of antiviral drugs, such as oseltamivir (Tamiflu^®^) and zanamivir, both of which target the enzymatic activity of NA and are effective against both influenza A and B virus strains. Mutations that render NA resistant to the aforementioned drugs have been reported. While NA is also capable of undergoing antigenic changes, these changes occur at a much slower rate than those observed with HA (Kilbourne et al., 1990). Given the relatively conserved nature of NA, there have been several potential vaccine candidates developed that target NA in order to generate improved influenza vaccines.

Unlike HA antibodies, NA antibodies do not neutralize infection, but they have been shown to inhibit NA enzymatic activity as well as reduce viral titers in mouse models (Rott et al., 1974). In addition, NA antibodies have been shown to be protective in both chickens and humans (Murphy et al., 1972; Clements et al., 1986; Webster et al., 1988). In humans, antibodies against NA have been associated with decreased viral shedding and shortened duration of symptoms (Maier et al., 2019). Recent studies have highlighted the importance of examining neuraminidase inhibition titers as well as hemagglutinin inhibition titers as a measure of influenza disease severity, suggesting that NA-based protection should be an important consideration when developing new influenza vaccines (Monto et al., 2015; Memoli et al., 2016).

There have been several approaches taken to develop NA-based vaccines, including use of recombinant NA proteins, DNA plasmid-based NA expression, and NA incorporation onto VLPs to boost NA-directed antibody responses (Sandbulte et al., 2007; Easterbrook et al., 2012; Liu et al., 2015; Job et al., 2018). Recently, a recombinant modified vaccinia virus Ankara (MVA) vector was used to express either HA or NA from three different H7 viruses. Following vaccination with a MVA vector expressing N3 NA, mice were protected against challenge with H7N3 (Meseda et al., 2018). Likewise, a passive transfer of sera from MVA-N3 vaccinated mice into naive mice demonstrated protection against H7N3 infection (Meseda et al., 2018) In contrast, mice that received sera from the MVA-N7 vaccine were not protected against challenge with H7N3 despite the vaccine containing an NA from the same subtype as N3, suggesting that further optimization of this vaccine approach is required to elicit broader NA-based protection (Meseda et al., 2018). Another NA vaccine approach involved the use of recombinant baculovirus VLPs expressing the N1 NA from A/California/04/2009 (N1 VLP) (Kim et al., 2019). The N1 VLP vaccine elicited NA inhibition activity for H1N1, H5N1, and H3N2 (Kim et al., 2019). Additionally, the N1 VLP vaccine provided protection in mice against challenge with H1N1, H5N1, and H3N2 (Kim et al., 2019). This suggests that the N1 VLP vaccine has the potential to confer protection against different influenza strains with different N1 subtype NAs (Kim et al., 2019). Additional studies in animal models that more closely recapitulate human influenza infection, like ferrets, are necessary to further examine the efficacy of this vaccine, but this study does provide more evidence for the importance of considering NA-based immune responses for influenza vaccines.

An important challenge in inducing NA directed immune responses involves overcoming the immunodominance of HA over NA. In an attempt to subvert the HA immunodominance, a recent study generated two recombinant influenza viruses based on the H1N1 stain A/Puerto Rico/8/1934 (PR8) in which the NA stalk domain was extended by 15–30 amino acids (Broecker et al., 2019b). Using formalin-inactivated viruses expressing wildtype NA or the extended NA, the authors demonstrated that the extended NA stalk induced higher anti-NA IgG responses than the unmodified NA in mice (Broecker et al., 2019b). Similarly, extension of the NA stalk from H3N2 virus increased NA-specific antibody responses (Broecker et al., 2019b). While additional challenge studies need to be performed, the NA stalk extension approach offers an interesting solution to improving the immunogenicity of NA.

As with HA-directed vaccination approaches, these NA-directed approaches do not always demonstrate that protection elicited by the vaccine extends to both NA groups. Another consideration for NA-directed vaccines involves whether these approaches can provide robust protection in humans. As stated previously, NA-directed antibodies do not neutralize infection, and antibodies against NA have been shown to reduce the duration of symptoms in humans. As a result, more studies demonstrating protection in humans or models that more accurately reflect human influenza virus infection are necessary to demonstrate the protection these NA-based methods can provide.

### M2 Ectodomain-Directed

The third surface protein on the influenza virion is the M2 protein. M2 is encoded by the M segment, which encodes M1 from unspliced mRNA and M2 protein by mRNA splicing (Fields et al., 2013). M2 forms homotetramers and possesses ion channel activity that allows for acidification of the inside of the virion during endocytosis and facilitates the dissociation of the matrix protein M1 from viral ribonucleoprotein complexes (Fields et al., 2013). The M2 ectodomain (M2e), which is the exposed portion of the M2 protein found on the virion membrane, is highly conserved among influenza strains (Ito et al., 1991). Additionally, M2e-directed antibodies have been detected during influenza infection and have been shown to be protective in both mice and ferrets (Black et al., 1993; Neirynck et al., 1999; Zharikova et al., 2005; El Bakkouri et al., 2011). Importantly, like NA-directed antibodies, M2 antibodies do not prevent infection but instead reduce disease severity and control the spread of infection (Mozdzanowska et al., 1999; Grandea et al., 2010). Given the conserved nature of M2e and the protection demonstrated with M2e-directed antibodies, M2e has become a target for universal influenza vaccine approaches.

As with NA and the HA stalk, M2e-directed immune responses must overcome the highly immunogenic HA head domain. In addition, while M2 is abundantly expressed on the cell surface of infected cells, M2 is less abundant on the virion itself, a challenge for eliciting robust immune responses in LAIV approaches due to the reduced availability of M2 on the LAIV virion (Lamb et al., 1985; Zebedee and Lamb, 1988). Previous attempts to improve M2e-directed immune responses involved the use of several viral vectors including papaya mosaic virus (PapMV) carrying M2e, HPV VLPs conjugated to the M2 protein, or VLPs derived from the RNA phage Qβ that display the M2e protein (Ionescu et al., 2006; Bessa et al., 2008; Denis et al., 2008). These M2e-based vaccines elicited M2 specific antibody responses and protected mice against influenza A virus challenge, highlighting the importance of M2e mediated protection and the importance of considering the M2e protein when formulating universal influenza vaccines (Ionescu et al., 2006; Bessa et al., 2008; Denis et al., 2008). Another approach to improve M2e antibody responses involved expressing a membrane anchored tandem repeat of M2e epitope sequences of human, swine, and avian origin on recombinant baculovirus VLPs (M2e5x VLP) (Kim et al., 2013; Kang et al., 2019). The M2e5x VLPs induced M2e-specific antibody responses against H1N1, H3N2, and H5N1 and conferred protection in mice against both H1N1 and H3N2 challenges (Kim et al., 2013). Recently, combinatorial vaccination with both M2e5x VLPs and HA-VLPs generated higher antibody titers, reduced lung inflammation, and provided improved protection against lethal challenge with H5N1 as compared to M2e5x VLP alone (Kang et al., 2019). While this study did not address whether these responses were greater than with HA-VLP vaccination alone, this study demonstrated the utility of combinatorial vaccination approaches to induce broader protection. The M2e5x approach was taken a step further by supplementing an attenuated pandemic A/Netherlands/602/09 LAIV with M2e5x VLPs (Lee et al., 2019). This strategy showed improved protection from morbidity and mortality in mice following viral challenge as compared to vaccination with LAIV alone (Lee et al., 2019). Importantly, incorporating LAIV into this approach allowed for induction of T-cell responses, providing an additional layer of protection against influenza viruses in the respiratory tract (Lee et al., 2019). As with NA-directed approaches, the question remains whether M2e vaccines are able to provide meaningful protection in humans when M2e directed antibodies are only shown to reduce disease severity. Likewise, the modest protection provided by M2e vaccines is a limitation for this vaccination approach as is the breadth of influenza strain protection. While M2e vaccination approaches may not provide as strong of protection as other methods, an exciting possibility for M2e approaches involves the inclusion of M2e into other vaccine strategies. This approach should be considered in the development of other vaccines to enhance the immunogenicity and protection against a broader range of influenza viruses.

### T-Cell-Directed

In comparison to the surface glycoproteins of influenza virus, the internal proteins show higher degrees of conservation among influenza viruses and are often targeted by the antigen-specific CD8^+^ cytotoxic T cells and CD4^+^ helper T lymphocytes. Given the importance of T cells in protection against influenza virus infection, vaccines stimulating influenza specific T-cell immunity have been explored as a promising avenue for improving influenza vaccine efficacy and developing a universal influenza vaccine.

Most T-cell-directed vaccine approaches involve targeting conserved internal influenza proteins or other highly conserved epitopes that stimulate T-cell-mediated immune responses. One such vaccine approach involved recombinantly expressing one B-cell epitope and two T-cell epitopes from H3 influenza strains in the flagellin of the *Salmonella* vaccine strain (Ben-Yedidia et al., 1999). After demonstrating the majority of the transplanted human cells in their human/mouse radiation chimeras were CD8^+^ and CD4^+^ cells, the recombinant vaccine was shown to elicit virus-specific antibodies and improved viral clearance after lethal challenge (Ben-Yedidia et al., 1999). In order to develop a more broadly protective vaccine, another study sought to identify conserved T-cell reactive regions by analyzing sequences from human and zoonotic influenza A and B viral proteins (Stoloff and Caparros-Wanderley, 2007). The internal proteins M1, NP, and PB1 and the surface protein M2 were found to contain conserved T-cell reactive regions, with M2 and PB1 sharing conserved sequences in both influenza A and B isolates (Stoloff and Caparros-Wanderley, 2007). Mice immunized with an antigen preparation comprised of the six conserved T-cell reactive regions, named FLU-v displayed increased CD8^+^ T-cell responses and improved survival following challenge with PR8 (Stoloff and Caparros-Wanderley, 2007). While this study did not determine whether the vaccine provides protection against challenge with influenza B infection, it does further demonstrate the importance of stimulating CD8^+^ T cells to generate protection against influenza infection. This vaccine is currently undergoing phase 2 clinical trials (Bernstein et al., 2019). Another conserved epitope vaccine undergoing clinical trials involving conserved T-cell and B-cell epitopes is the Multimeric-001 vaccine, which consists of nine conserved epitopes of HA, NP, and M1 from influenza A and B viruses (Atsmon et al., 2012). Healthy human volunteers vaccinated with the Multimeric-001 vaccine exhibited increased IgG titers against the Multimeric-001 vaccine component as well as increased IL-2 and IFNγ secretion (Atsmon et al., 2012). Additionally, sera from vaccinated subjects showed increased complement mediated lysis of infected MDCK cells (Atsmon et al., 2012). This measure was incorporated as an alternative to the hemagglutinin inhibition assay, which they were unable to use because the vaccine lacked the antigenic sites for neutralizing antibodies (Atsmon et al., 2012). While this study shows promise for inducing broad protection in humans, further challenge studies are needed to determine the efficacy of this vaccine.

As with the other vaccine approaches, there have been attempts to generate T-cell-based influenza vaccines through the use of viral vectors. One approach currently in clinical trials involves the use of a modified vaccinia virus Ankara (MVA) that encodes influenza proteins NP and M1 (MVA – NP + M1) (Berthoud et al., 2011; Lillie et al., 2012). A phase 1 clinical trial demonstrated that individuals vaccinated with the MVA – NP + M1 vaccine exhibited increased T-cell responses as measured by IFNγ ELISPOT, with the majority of them being antigen-specific T cells (Berthoud et al., 2011). Next, a phase 2a clinical trial was conducted in which volunteers vaccinated with the MVA – NP + M1 exhibited less severe symptoms and reduced viral shedding after challenge with A/Wisconsin/67/2005 (H3N2) (Lillie et al., 2012). This study was performed with a limited number of human volunteers, but it does highlight another promising avenue for stimulating T-cell-based protection against influenza.

Recently, another viral vector-based vaccine approach has been described using the live-attenuated vaccinia Wyeth backbone expressing HA, NA, M1, M2, and NP from H5N1 along with IL-15 as an adjuvant (Poon et al., 2009). In a mouse model, this vaccine was shown to be protective against both Group 1 and Group 2 HA viruses, including H7N9, H3N2, H1N1, and H7N7 (Valkenburg et al., 2014). Following depletion of CD4^+^ T cells at the time of vaccination or challenge, vaccinated mice showed reduced survival, suggesting that CD4^+^ T cells are required for this vaccine-mediated protection (Valkenburg et al., 2018). This vaccine also stimulated increased production of H5-specific CD4^+^ and CD8^+^ T cells from human PBMCs, highlighting its potential for protection in humans as well (Valkenburg et al., 2018). Together, these studies illustrate the importance of stimulating T-cell-based immunity for influenza virus protection.

### Challenges to Universal Vaccine Development

An important question when discussing universal vaccine development involves defining the criteria for a successful universal influenza vaccine. Would a universal vaccine provide protection against all influenza A and B strains? Only influenza A or B? Or would the vaccine only provide universal protection within particular HA or NA subtypes? The route of administration should also be considered, especially when determining the durability of protection provided by the vaccine. Furthermore, many of the universal vaccine strategies described above require sequential vaccinations or boosters in order to achieve protection. Considering that individuals may be less likely to return to complete their vaccination regimens, this approach may have a negative impact on vaccine effectiveness from a public health standpoint. Likewise, sequential vaccination regimens with components that change at each booster vaccination may increase the likelihood of error during administration.

While the universal vaccine approaches described above demonstrate novel methods of improving protection against influenza virus infection, a concern that is often overlooked involves the degree to which pre-existing immunity impacts the antibody response to influenza infection and vaccination. This is true for all of the vaccination approaches, including HA, NA, M2e, and T-cell-directed approaches. This concept, referred to as “original antigenic sin,” suggests that the first influenza virus variant an individual encounters impacts the immune response to subsequent influenza virus variants (Henry et al., 2018). While controversial, the concept that previous influenza virus exposure impacts antibody responses to influenza vaccination remains an important consideration for vaccine development (Henry et al., 2018). This can also be an important determinant in LAIV approaches, where pre-existing immunity can impact LAIV replication. It is important to note that most of the animal studies done to validate these vaccination approaches are done in naïve animals that lack any previous influenza virus exposure. While unavoidable, this serves to highlight the importance of experiments that more accurately recapitulate the complexity of pre-existing antibody responses in humans as well as thorough clinical trials in order to fully assess the potential for new vaccines to provide broad and long-lasting protection within the population.

## Therapeutic Approach: Broadly Neutralizing Antibodies

While vaccination remains the most efficient method to provide protection against influenza virus infection, there is also significant interest in developing more broadly protective therapeutic methods for prevention and treatment of influenza virus infection. A growing area of interest involves utilizing broadly neutralizing antibodies to protect against influenza virus infection. As with influenza vaccines, a significant focus on broadly neutralizing antibody research revolves around the development of HA stem targeting antibodies, in particular antibodies that neutralize both Group 1 and Group 2 influenza virus HAs. Despite the fact that the majority of neutralizing antibodies targeting the HA stem only protect against Group 1 or Group 2 HAs, HA targeting antibodies capable of neutralizing both phylogenetic groups have been identified (Corti et al., 2011; Joyce et al., 2016). Further improvements in broadly neutralizing antibody discovery have enabled the development of novel therapies, several of which are currently in different stages of clinical trials. For example, one study was able to identify four broadly neutralizing influenza A antibodies through the activation and enrichment of human peripheral blood mononuclear cells from vaccinated donors (Nakamura et al., 2013). Of these four antibodies, two were able to neutralize both Group 1 and Group 2 influenza A viruses while also improving the survival of mice and ferrets that received the antibody 72 h post infection (Nakamura et al., 2013). Interestingly, co-administration of oseltamivir and the antibody after lethal challenge significantly improved the survival of mice as compared to either therapy alone, demonstrating a potential avenue for improving current therapies through co-administration of both antivirals and neutralizing antibodies (Nakamura et al., 2013). Using a novel antibody design approach, another broadly neutralizing HA antibody, VIS410, was engineered and shown to bind both Group 1 and Group 2 HAs (Tharakaraman et al., 2015). Experiments in mice demonstrated that VIS410 improved survival when administered either prophylactically or 48 h post infection (Tharakaraman et al., 2015). These studies also demonstrated improved protection when co-administered with oseltamivir (Tharakaraman et al., 2015). When administered post infection, VIS401-treated mice showed reduced clinical symptoms of influenza virus infection, including decreased viral spread and reduced damage in the lungs (Baranovich et al., 2016). VIS401 is currently under clinical trials. MEDI8852 is another broadly neutralizing antibody targeting the HA stem that has been shown to interact with both Group 1 and Group 2 HAs (Kallewaard et al., 2016). Even when administered 4 days post lethal virus challenge, MEDI8852 treatment improved survival of mice and protected against several different influenza A virus strains (Kallewaard et al., 2016). Similar results were observed in ferrets, with MEDI8852 providing protection against lethal challenge when administered up to 3 days post infection, further underscoring the therapeutic potential of this antibody (Kallewaard et al., 2016). In addition to several HA targeting monoclonal antibodies, a M2e targeting antibody, TCN-032, is currently in clinical trials (Ramos et al., 2015). Healthy human volunteers infected with H3N2 and treated with TCN-032 exhibited reduced viral shedding and reduced clinical symptoms (Ramos et al., 2015). While the broadly neutralizing antibodies in these studies show promise in providing protection in humans, the studies fail to address the potential for the development of escape mutants that are able to evade antibody neutralization following treatment with these antibodies. Studies examining whether prolonged treatment with these antibodies leads to resistance are important in determining whether these therapeutics can truly provide meaningful protection for a wide range of individuals. Together, these studies underscore the potential for utilizing broadly neutralizing antibodies to improve clinical outcomes of influenza virus infection.

## Conclusions

Significant resources have been invested into the development of universal influenza virus vaccines and improved therapeutics for the treatment of influenza virus infection. Many universal influenza virus vaccine candidates focus on targeting conserved epitopes of influenza, including either the three influenza surface proteins or highly conserved internal proteins. In doing so, these approaches attempt to elicit broader protection against several strains of influenza as opposed to the strain-specific protection provided by current seasonal influenza vaccines. In attempting to generate a truly universal vaccine, which would offer protection against all influenza strains, these approaches have also highlighted innovative methods for improving current vaccination approaches. By providing protection for a broader range of influenza strains, these approaches have the potential to reduce the need for yearly vaccine reformulations. While influenza strains should still be closely monitored, broader protection against more influenza strains could aid in improving yearly vaccine efficacy. The development and discovery of broadly neutralizing antibodies also illustrate the important contributions these therapeutics can offer in preventing and improving disease outcomes associated with influenza virus infection. Additionally, these studies have provided an insight into novel combinatorial approaches, as seen with studies combining M2e5x VLPs and HA-VLPs to improve vaccine immunogenicity or by combining broadly neutralizing antibodies with current antivirals to improve recovery from influenza virus infection (Nakamura et al., 2013; Tharakaraman et al., 2015; Kang et al., 2019). While the ultimate goal of developing a truly universal influenza virus vaccine has yet to be achieved, the progress made in pursuit of this goal shows the exciting promise of these new approaches for improving influenza disease outcomes and as well as the public health burden associated with inefficient protection against influenza virus infection.

## Author Contributions

All authors listed have made a substantial, direct and intellectual contribution to the work, and approved it for publication.

### Conflict of Interest

The authors declare that the research was conducted in the absence of any commercial or financial relationships that could be construed as a potential conflict of interest.
